# GATA3 Positively Correlates with BCL2 Expression in Indolent and Aggressive Histological Types of Cutaneous Basal Cell Carcinoma

**DOI:** 10.5146/tjpath.2024.13370

**Published:** 2024-09-02

**Authors:** Fatma Alzahraa Abdelsalam Elkhamisy, Ahmed Naeem Eesa, Marwa Kamal Sallam, Marwa Fathy Hussein, Ahmed Abd El-Moeze

**Affiliations:** Department of Pathology, Helwan University, Faculty of Medicine, Cairo, Egypt; Cairo University, Faculty of Medicine, Giza, Egypt; Department of Medical Microbiology and Immunology, Faculty of Medicine and Kasr Al Ainy Hospitals, Cairo University, Giza, Egypt; Department of Dermatology, Cairo University, Faculty of Medicine, Giza, Egypt; Department of Pathology, Beni-Suef University, Faculty of Medicine, Beni-Suef, Egypt

**Keywords:** Basal cell carcinoma, BCL2, GATA3, Tumor-infiltrating lymphocytes, Tumor aggressiveness

## Abstract

***Objective: *** Some histological basal cell carcinoma (BCC) types demonstrate more aggressive behavior than others. They are known as high-risk BCC and are more challenging in therapy, contrary to indolent (low-risk) BCC types. Identifying novel protein markers to predict aggressiveness and potential therapeutic targets in challenging cases is recommended. GATA3 is a transcription factor critical for epithelial and lymphocytic differentiation. This study investigated the immunohistochemical expression of GATA3 in indolent and aggressive BCC and its association with BCL2 expression.

***Material and Methods:*** Retrospectively collected indolent and aggressive BCC groups (24 cases each) were immunohistochemically stained with anti-GATA3 and BCL2 antibodies. The mean expression score (by area percentage) and TIL counts were determined and compared using ImageJ analysis. Stromal tumor-infiltrating lymphocytes (TIL) were counted per high-power field (HPF) on hematoxylin and eosin (H&E) staining.

***Results: *** GATA3 and BCL2 expressions were significantly higher in the indolent group than in the aggressive group. GATA3 expression significantly correlated with BCL2 score and TIL counts. Higher GATA3 expression was significantly associated with a more indolent BCC histological type, higher BCL2 expression, and higher TIL count.

***Conclusion:*** GATA3 is a possible target for immunomodulation experiments to improve BCC immunotherapy outcomes.

## Introduction

Basal cell carcinoma (BCC) is the most common non-melanoma skin cancer worldwide, and its annual incidence is increasing (1,2). Despite its low mortality and extremely low metastatic rates, it has a high morbidity rate with local destruction and recurrence (3). Recently, there has been an investigation into the use of adjuvant and alternative local and systemic treatments for challenging BCC cases, including multiple, locally advanced, and metastatic tumors (1,4).

The likelihood of aggressive tumor behavior, including depth of invasion, recurrence, and local and distant metastases, determines the classification of the BCC histopathological subgroups. BCCs with a low risk of aggressiveness (i.e., indolent behavior) include nodular, superficial, pigmented, infundibulocystic (BCC with adnexal differentiation), adenoid, and fibroepithelial types. In contrast, micronodular, infiltrating, sclerosing/morphoeic, basosquamous, and BCC with sarcomatoid differentiation have a significant risk of aggressive behavior (1).

Identifying new BCC protein targets will open the door to investigate new treatment modalities. Some immunohistochemical (IHC) markers have been associated with BCC behavior. In many studies, reduced BCL2 expression has been associated with more aggressive BCC types; BCL2 is an essential modulator of the mitochondrial apoptotic pathway, which promotes cell survival without increasing cell proliferation (5,6).

GATA3 is a zinc finger nuclear transcription factor. It activates or inhibits the activity of target genes by binding to G-A-T-A nucleotide sequences in the promoter regions (7). It plays a pivotal role in T lymphocyte development and differentiation as well as in epithelial tissue differentiation (8). Breast, urothelial, and BCC cancers have the highest GATA3 expression; the limited expression of GATA3 in specific tissues has made it useful as a diagnostic IHC marker in cancers in these tissues (9).

An association between GATA3 expression and cancer aggressiveness has also been previously reported. As a tumor suppressor, high expression is associated with promising prognostic findings in some cancers, including breast and urothelial (10). However, it acts as an oncogene and is associated with poor prognosis in other cancers, such as esophageal squamous cell carcinoma (SCC) (11). GATA3 expression positively correlates with BCL2 expression in some tumors (12). Many reports have investigated GATA3 expression in BCC as a possible diagnostic marker and have reported strong expression (7,9). However, no published studies have investigated the association between GATA3 expression and BCC aggressiveness or BCL2 expression.

Understanding the role of GATA3 in BCC by studying its association with BCC aggressiveness will help to understand BCC pathogenesis further and open the door to further research areas and possible new treatment opportunities involving the modulation of the GATA3 pathway. Therefore, in this study, we aimed to investigate GATA3 expression in indolent and aggressive histological BCC and its correlation with BCL2 expression. We hypothesized that GATA3 is associated with BCC behavior and BCL2 expression.

## Materials and methods

### Study Design, Data, and Specimen Collection

The study was a retrospective, comparative, and cross-sectional study, in which IHC GATA3 and BCL2 co-expression was compared between two BCC groups: histologically indolent and aggressive. Surgically excised BCC specimens were retrospectively collected from the Pathology Laboratory at the Specialized Medical Center, Faculty of Medicine, Beni-Suef University, Egypt, between January 2019 and December 2022. The collected specimens were formalin-fixed and paraffin-embedded. Available clinical and pathological data, including the patient’s age at diagnosis, sex, tumor site, and tumor size, were recorded from the cases’ pathology requests and reports.

The sample size was calculated using the STATA program, setting the type-1 error (α) at 0.05 and the power (1-β) at 0.9. A pilot study on ten patients per group showed that the mean GATA3 among indolent cases was 10.9±2.6 compared to 8.3±2.4 in aggressive cases. Calculations based on these values yielded a sample size of 21 cases per group. The study included 24 cases in each group to compensate for tissue dropouts during the processing of slides (48 cases in total). All surgically excised BCC cases retrieved during the specified specimen collection duration of the study were included. Samples were excluded if there was insufficient or over-fixed material or artifacts in the process.

### Histopathologic Evaluation

Two consultant pathologists independently examined the H&E-stained BCC slides to confirm the diagnosis, determine the histological type, and report any other positive, relevant histological findings like the presence/absence of perineural and surgical margin invasion. The cases were grouped into indolent and aggressive groups according to histological BCC types. In tumors with mixed histological subtypes, predominant components were recorded. In addition, quantitative image analysis (Image J 1.53t, Wayne Rasband and contributors, National Institutes of Health, USA) was used on H&E slides to count the TIL in BCC stroma and calculate the mean count for ten randomly selected high-power (400x) fields in each section. Stromal TILs are lymphocytes scattered in the stroma between cancer cells, without direct contact with cancer cells (13).

### Immunohistochemical Examination

Four μm thick sections from each representative tumor block were mounted on positively charged slides using the avidin-biotin-peroxidase complex (ABC) method and subjected to IHC staining with monoclonal rabbit GATA3 antibody (Cell Signaling Technology Cat# 13411, RRID: AB_2798212), dilution 1:1600, and polyclonal rabbit BCL2 antibody (Novusbio, Cat# NBP2-30108), dilution 2 μg/ml. The staining protocols for the anti-GATA3 and BCL2 products were performed according to the manufacturer’s instructions. The reagents required for the ABC method were added (Vectastain ABC-HRP Kit; Vector Laboratories). Marker expression was labeled with peroxidase and stained with diaminobenzidine (DAB, Sigma) to detect the antigen-antibody complex. All slide-processing procedures included both positive and negative controls. The omission of the primary antibody was used as a negative control for non-specific staining with a secondary antibody (14). Tonsils were used as a negative control for GATA3 and BCL2 markers, with non-stained B-cell areas for GATA3 and non-stained germinal centers for BCL2.

Positivity was determined by observing all slides at a low power (40×) and then randomly selecting ten fields at a high power (400×) to estimate the average percentage of immunolabel-positive cells. Several areas were examined to increase the reproducibility of the results (14). Stained sections were scored as positive if > 0% of malignant cells showed nuclear staining for GATA3 and cytoplasmic or nuclear BCL2 staining.

The IHC-stained sections were visualized under an Olympus microscope (BX-53). Automated quantitative scoring of marker expression was performed to avoid the reported shortcomings of manual scoring regarding reproducibility (14,15). Image analysis was used to score the selected foci based on automatic analysis of the color staining intensity and reaction area, giving a percentage area score. The IHC-stained slides were scored independently by two different pathologists using the threshold method, and the mean of the two pathologists’ scores/10 HPFs/slide was reported. The inter-scorer difference between the two pathologists’ scores was statistically insignificant.

### Statistical Analysis

The collected data were revised, coded, tabulated, and introduced to a PC using Statistical Package for Social Science (IBM Corp. Released 2017. IBM SPSS Statistics for Windows, Version 25.0. Armonk, NY: IBM Corp). Shapiro Wilk’s test was used to evaluate the normal distribution of quantitative variables, expressed as mean and SD. For univariate analysis, Student’s t-test was used to compare a Quantitative variable between two study groups. Categorical variables were compared using the Chi-square and described in frequency (count) and percentage. Pearson’s Correlation analysis was used to assess the strength of the association between two variables. Multivariate linear Regression (MLR) analysis was used to determine factors associated independently with the outcome variable (GATA3). A P-value <0.05 was considered statistically significant, and <0.001 was considered highly statistically significant.

### Quality Measures

We followed the recommendations in the methodology for increasing the reproducibility of scoring results in tissue stains (14). Moreover, we used the Reporting Recommendations for Tumor Marker Prognostic Studies (REMARK) guidelines (16) to assess the quality of our article and enhance the possibility of comparing results among studies investigating molecular biomarkers.

### Compliance with Ethical Standards

The study received approval from the Faculty of Medicine Ethics Committee, Beni-Suef University, Egypt, IRB number: FMBSUREC/09072023. Formal written informed consent was not required with a waiver by the IRB research ethics committee. All work was carried out in compliance with the Helsinki Declaration of 1964 and its later amendments. All personal data of the cases were deidentified, and all cases were coded before further inclusion in the study.

## Results

Table 1 shows the clinicopathological features of the BCC cases in the indolent and aggressive groups. The aggressive BCC group appeared at a younger age, had a larger size evaluated by the mean diameter, and had a higher propensity to affect the scalp than the indolent group. However, there were no statistically significant differences in the mean age, sex, tumor size, or site of the BCC tumor between the indolent and aggressive groups (Table 1).

The most prevalent histological subtype in the indolent group was nodular (n=12, 50%), followed by an equal number of pigmented, adenoid, and superficial subtypes (n=4, 16.7% each). Among the aggressive group, basosquamous (metatypical) carcinoma was the most prevalent (n=14, 58.3%), followed by morphea (n=7, 29.2%) and micronodular subtypes (n=3, 12.5%). All specimens were margin-free and negative for perineural invasion.

**Figure 1:**
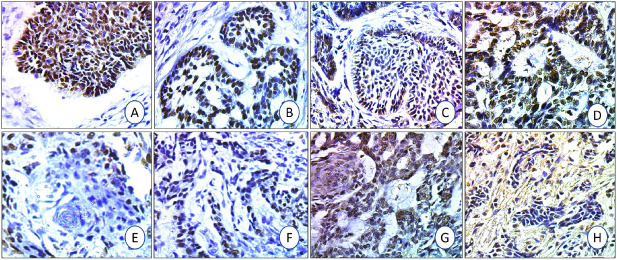
GATA3 and BCL2 immunohistochemical expression is higher in indolent compared to aggressive BCC. GATA3 (**A:** nodular & **B:** adenoid types) and BCL2 (**C:** nodular & **D:** adenoid types) expression in the indolent group. GATA3 (**E:** basosquamous & **F:** morphea types) and BCL2 (**G:** basosquamous & **H:** morphea types) expression in the aggressive BCC groups (X200).

All BCC cases showed positive GATA3 and BCL2 expressions. The expression was higher at the advancing edges of the tumor nodules. Highly statistically significant mean GATA3 and BCL2 scoring was observed in the indolent BCC group (12.95±3.26%/HPF, 18.42±3.22%/HPF), compared to the aggressive group (9.61±4.82%/HPF, 6.86±1.68%/HPF) (p=0.007, 0.0001) for GATA3 and BCL2, respectively (Table 1 and Figure 1).

**Table 1:** Clinicopathological characteristics and GATA3 and BCL2 expression in the indolent and aggressive BCC groups in the study.

	**Indolent**	**Aggressive**	***P*** **-value**
**Age (years)**			0.157^a^
Range	36-76	52-69	
Mean (±SD)	63.83 (±12.77)	59.75 (±5.51)	
**Sex, n (%)**			1.00^b^
Male	12 (50)	12 (50)	
Female	12 (50)	12 (50)	
**Site, n (%)**			0.182^b^
Face	20 (83.3)	16 (66.7)	
Scalp	4 (16.7)	8 (33.3)	
**Size (cm)**			0.096^a^
Range	1.00-3.60	1.30-4.10	
Size by Largest Diameter Mean (±SD)	2.36 (±0.82)	2.76 (±0.83)	
**Tumor-infiltrating lymphocyte count mean/HPF**			0.0001^a^
Range	189.70-440.60	87.30-374.10	
Mean (±SD)	378.97 (±70.59)	246.33 (±93.45)	
**GATA3 mean expression score/HPF**			0.007^a^
Range	8.73-21.67	3.27-21.01	
Mean (±SD)	12.95 (±3.26)	9.61 (±4.82)	
**BCL2 mean expression score/HPF**			0.0001^a^
Range	13.55-23.18	4.20-9.50	
Mean (±SD)	18.42 (±3.22)	6.86 (±1.68)	
**Total**	24 (100%)	24 (100%)	

^a^Student t-test; ^b^Chi-Square Test; HPF: High Power Field

**Figure 2:**
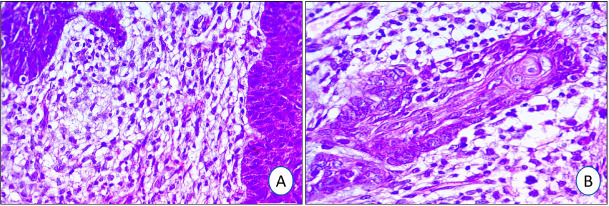
Stromal mononuclear inflammatory cellular infiltrate in BCC: Lymphocytes are higher in the indolent (**A:** nodular type) compared to the aggressive (**B:** basosquamous type) BCC on Hematoxylin and Eosin stain (X200)

The peritumoral BCC stroma showed an invariable mixture of mononuclear inflammatory cellular infiltrate. Compared to the stromal tumor-infiltrating lymphocyte (TIL) count in both BCC groups, the indolent group showed a significantly higher (p=0.0001) mean count (378.97±70.59 cells/HPF) than the aggressive group (246.33±93.45 cells/HPF), as shown in Table 1 and Figure 2.

Pearson’s correlation showed a statistically significant positive correlation between GATA3 expression and the mean TIL count (R=0.5, p=0.000) and between BCL2 expression and the mean TIL count (R=0.604, p=0.000). Furthermore, a statistically significant positive correlation existed between BCL2 and GATA3 expression (R=0.409, P=0.004).

Multivariate backward linear regression with adjustment for all studied variables showed that age, sex, tumor site, tumor size, TIL count, and BCL2 score were independent variables affecting GATA3 expression. An increase in age, female sex, and increasing tumor size were associated with lower GATA3 levels (p<0.001, p<0.05, p<0.001, respectively). While scalp tumors, the increase in stromal TILs and the increase in BCL2 expression were associated with higher GATA3 levels (p<0.001, p<0.001, p<0.001, respectively) (Table 2).

**Table 2:** The multivariate backward linear regression analysis to detect the independent variables affecting the GATA3 expression score.

	**Regression** **Coefficients (B)**	**P**	**95% Confidence Interval for B**	
			**Lower Bound**	**Upper Bound**
Age	-0.164	0.0001	-.244	-.084
Female (Gender)	-4.587	0.0001	-6.137	-3.037
Scalp (Site)	9.565	0.0001	7.341	11.788
Size	-1.909	0.002	-3.101	-.716
Tumor-Infiltrating Lymphocytes	0.011	0.015	.002	.020
BCL2	0.280	0.001	.129	.431

## Discussion

Some BCCs behave aggressively, with deep invasion, recurrence, and potential regional and distant metastases. More aggressive behavior is linked to several factors, including the histological BCC phenotype with higher subclinical extension, leading to management difficulties and a worse prognosis than other types (17). Specific expression patterns of IHC markers can indicate indolent and aggressive BCC behavior, aiding in the prediction of the prognosis as well as acting as possible therapeutic targets (1,6,18). Our study showed significantly higher GATA3 expression in the histologically indolent BCC compared to the aggressive group, with a significant positive correlation between GATA3 expression and BCL2 expression and TIL counts in BCC stroma, supporting our study hypothesis.

Many studies have reported high and robust GATA3 expression in BCC cases, reaching up to 97% and 98% in cases (7–9). Similarly, our study showed high positivity for GATA3, which reached 100% of the cases. This variation may be attributed to differences in staining protocols between studies and the relatively small number of cases in our study. In this study, higher GATA3 expression was associated with less aggressive (indolent) BCC. Higher GATA3 expression has been reported to be associated with good prognostic criteria in urothelial cancer, such as a lower tumor grade and stage (19), and in breast cancer, with better tumor differentiation and suppressed metastasis (20). Loss of GATA3 expression in breast cancer has been linked to poor prognosis with a higher tumor T stage, HER2 overexpression, estrogen and progesterone receptor negativity, and reduced survival (9). This association with good tumor behavior, including in BCC, can be explained by the crucial role played by GATA3 in developing epithelial structures in both embryonic and adult tissues and in promoting cell differentiation in many tissues, including skin (8).

Understanding the effect of GATA3 expression on the BCC TME can open doors to immunomodulation and improve the treatment results of immunotherapy. Cutaneous BCC is an immunologically cold tumor (21,22). Local treatment of BCC with immunotherapy is a novel line of treatment with conflicting results (1). In the past few years, studies have investigated BCC TME with a focus on the pattern of inflammatory cell infiltration to understand the factors that affect BCC prognosis and response to immunotherapy (22,23). We reported a significant positive correlation between GATA3 expression score and TIL count in the BCC stroma. GATA3 is crucial for early T-cell commitment, the β-selection checkpoint before expressing a functional cell-surface pre-TCR, and CD4+ T-cell development (24). GATA3 is a master regulator of T-helper 2 (Th2)-cell differentiation (8).

In contrast to our results, high GATA3 expression is associated with low scores of infiltrating lymphocytes in the tumor microenvironment (TME) of bladder cancer (25). The varying composition of stromal lymphocytes in tumors can explain this difference. The composition of BCC-TILs is still poorly understood. Some studies have shown a dominant downregulation of the CD4 and CD8 immune response (23) and a dominant Treg suppressor cell component (26). In some reports, in cases of spontaneous BCC regression, the BCC stroma has shown very high TILs that reached above 950 cells/cm2/HPF (27). In these cases, TIL cells dominated inflammatory Th or T Cytotoxic (Tc) types (27,28), with a low percentage of Treg phenotype (27). Future research comparing the TIL composition in aggressive and non-aggressive BCC and its relation to GATA3 expression is required to understand better GATA3’s role in the BCC TME and BCC response to immunotherapy.

Similar to other studies, we reported significantly higher BCL2 expression in histologically indolent BCC than in aggressive BCC groups (5,6). Although BCL2 is an oncogene, it prolongs epithelial cell lifespan and reduces apoptosis without stimulating cell proliferation (5). This survival advantage permits differentiation potential and morphogenesis, and explains its high expression in some low-grade neoplasms, including BCCs (5).

GATA3 expression was significantly positively correlated with BCL2 expression in our study, and both were higher in the indolent group. Similarly, GATA3 expression was positively correlated with BCL2 expression in breast cancer (12). The interplay between BCL2 and GATA3 expression has still not been fully explained in the literature, and further studies are needed to investigate the precise common pathways. Some authors have suggested that GATA3 might regulate BCL2 transcription and, hence, its expression directly by binding to the promoter region of BCL2 or might indirectly affect its transcriptional regulatory effect on other genes and pathways (12,29).

In conclusion, higher GATA3 expression was significantly associated with a more indolent BCC histological type, higher BCL2 expression, and a higher stromal tumor-infiltrating lymphocyte count. GATA3 may serve as a potential target for immunomodulation experiments to enhance the outcomes of BCC immunotherapy.

This study is the first to investigate GATA3 expression in histologically aggressive and indolent BCC groups and the first to investigate its association with BCL2 expression. Strengths also include using quantitative scoring through image analysis to eliminate subjectivity and to produce a higher dynamic range of captured data for better analysis than visual and quantitative categorical scores (15). In addition, selecting an increased number of HPFs (ten) in each section to score IHC marker expression results in higher accuracy for the marker’s mean score (16).

Study limitations included a cross-sectional study of available BCC cases; therefore, not all histological BCC variants were sampled. In addition, the limited number of cases and the fact that this was a single-institutional study might have affected the generalizability of the findings. Further research with more BCC cases and subtypes is required to validate the reported results. In addition, future research is recommended to investigate GATA3 expression in BCC with clinicopathological factors associated with higher aggressive behavior - other than the histological type - including different BCC locations and perineural invasion status.

## Data Availability

The data supporting this study’s findings are available from the corresponding author upon reasonable request.

## Funding

This research did not receive any specific grant from public, commercial, or not-for-profit funding agencies.

## Conflict of Interest

The authors declare that they have no potential conflicts of interest to disclose.

## References

[ref-1] Niculet Elena, Craescu Mihaela, Rebegea Laura, Bobeica Carmen, Nastase Florentina, Lupasteanu Gabriela, Stan Daniela Jicman, Chioncel Valentin, Anghel Lucretia, Lungu Mihaela, Tatu Alin Laurentiu (2022). Basal cell carcinoma: Comprehensive clinical and histopathological aspects, novel imaging tools and therapeutic approaches (Review). Exp Ther Med.

[ref-2] El-Khalawany Mohamed, Hassab-El-Naby Hussein M. M., Mousa Ahmed Mustafa, Sameh Ahmed, Rageh Mahmoud A., Genedy Rasha Mahmoud, Hosny Aya Magdy, Aboelmagd Marwa A., Aboeldahab Soha (2023). Epidemiological and clinicopathological analysis of basal cell carcinoma in Egyptian population: a 5-year retrospective multicenter study. J Cancer Res Clin Oncol.

[ref-3] Laga Alvaro C., Schaefer Inga Marie, Sholl Lynette M., French Christopher A., Hanna John (2019). Metastatic Basal Cell Carcinoma. Am J Clin Pathol.

[ref-4] Fredman Gabriella, Wenande Emily, Hendel Kristoffer, Togsverd-Bo Katrine, Haedersdal Merete (2022). Efficacy and safety of laser-assisted combination chemotherapy: A follow-up study of treatment with 5-fluorouracil and cisplatin for basal cell carcinoma. Lasers Surg Med.

[ref-5] Crowson A. N., Magro C. M., Kadin M. E., Stranc M. (1996). Differential expression of the bcl-2 oncogene in human basal cell carcinoma. Hum Pathol.

[ref-6] Iljin Aleksandra, Stasikowska-Kanicka Olga, Zieliñski Tomasz, Bąkiewicz Anna, Sporny Stanisław, Woźniak-Roszkowska Ewa, Antoszewski Bogusław (2022). Immunoexpression of Bmi-1, CK15, Bcl-2 in different types of basal cell carcinomas. Postepy Dermatol Alergol.

[ref-7] Mertens Richard B., Peralta-Venturina Mariza N., Balzer Bonnie L., Frishberg David P. (2015). GATA3 Expression in Normal Skin and in Benign and Malignant Epidermal and Cutaneous Adnexal Neoplasms. Am J Dermatopathol.

[ref-8] Khazaeli Najafabadi Mahdis, Mirzaeian Elham, Memar Montazerin Sahar, Tavangar Amir Reza, Tabary Mohammadreza, Tavangar Seyed Mohammad (2021). Role of GATA3 in tumor diagnosis: A review. Pathol Res Pract.

[ref-9] Reiswich Viktor, Schmidt Carol E., Lennartz Maximilian, Höflmayer Doris, Hube-Magg Claudia, Weidemann Sören, Fraune Christoph, Büscheck Franziska, Möller Katharina, Bernreuther Christian, Simon Ronald, Clauditz Till S., Blessin Niclas C., Bady Elena, Sauter Guido, Uhlig Ria, Steurer Stefan, Minner Sarah, Burandt Eike, Dum David, Marx Andreas H., Krech Till, Lebok Patrick, Hinsch Andrea, Jacobsen Frank (2023). GATA3 Expression in Human Tumors: A Tissue Microarray Study on 16,557 Tumors. Pathobiology.

[ref-10] Serag Eldien Marwa Mohammed, Abdou Asmaa Gaber, Elghrabawy Gehad Raafat Amein, Alhanafy Alshimaa Mahmoud, Mahmoud Shereen Fathy (2021). Stratification of urothelial bladder carcinoma depending on immunohistochemical expression of GATA3 and CK5/6. J Immunoassay Immunochem.

[ref-11] Chi Zhikai, Balani Jyoti, Gopal Purva, Hammer Suntrea, Xu Jing, Peng Lan (2023). GATA3 positivity is associated with poor prognosis in patients with oesophageal squamous cell carcinoma. J Clin Pathol.

[ref-12] Fararjeh Abdul-Fattah Salah, Tu Shih-Hsin, Chen Li-Ching, Liu Yun-Ru, Lin Yen-Kuang, Chang Hang-Lung, Chang Hui-Wen, Wu Chih-Hsiung, Hwang-Verslues Wendy W., Ho Yuan-Soon (2018). The impact of the effectiveness of GATA3 as a prognostic factor in breast cancer. Hum Pathol.

[ref-13] Salgado R., Denkert C., Demaria S., Sirtaine N., Klauschen F., Pruneri G., Wienert S., Eynden G., Baehner F. L., Penault-Llorca F., Perez E. A., Thompson E. A., Symmans W. F., Richardson A. L., Brock J., Criscitiello C., Bailey H., Ignatiadis M., Floris G., Sparano J., Kos Z., Nielsen T., Rimm D. L., Allison K. H., Reis-Filho J. S., Loibl S., Sotiriou C., Viale G., Badve S., Adams S., Willard-Gallo K., Loi S., International TILs Working Group 2014 (2015). The evaluation of tumor-infiltrating lymphocytes (TILs) in breast cancer: recommendations by an International TILs Working Group 2014. Ann Oncol.

[ref-14] Meyerholz David K., Beck Amanda P. (2018). Principles and approaches for reproducible scoring of tissue stains in research. Lab Invest.

[ref-15] Ram Sripad, Vizcarra Pamela, Whalen Pamela, Deng Shibing, Painter Cl, Jackson-Fisher Amy, Pirie-Shepherd Steven, Xia Xiaoling, Powell Eric L. (2021). Pixelwise H-score: a novel digital image analysis-based metric to quantify membrane biomarker expression from immunohistochemistry images.

[ref-16] McShane L. M., Altman D. G., Sauerbrei W., Taube S. E., Gion M., Clark G. M., Statistics Subcommittee of the NCI-EORTC Working Group on Cancer Diagnostics (2005). REporting recommendations for tumour MARKer prognostic studies (REMARK). Br J Cancer.

[ref-17] Ariza Santiago A., Calderón Diana C., Aristizábal Juan C., Parra-Medina Rafael (2020). How Wide Should the Excision Margins for Facial Small Aggressive Basal Cell Carcinoma Be? Experience With 306 Cases. Dermatol Surg.

[ref-18] Rajabi Parvin, Heydarpoor Mitra, Maghsoudi Ahmadreza, Mohaghegh Fatemeh, Dehghani Mobarakeh Maryam (2019). The Study for Diagnostic Value of β-Catenin Immunohistochemistry Marker in Distinction of Aggressive and Non-Aggressive Basal Cell Carcinoma. Iran J Pathol.

[ref-19] Bernardo Carina, Monteiro Fátima L., Direito Inês, Amado Francisco, Afreixo Vera, Santos Lúcio L., Helguero Luisa A. (2021). Association Between Estrogen Receptors and GATA3 in Bladder Cancer: A Systematic Review and Meta-Analysis of Their Clinicopathological Significance. Front Endocrinol (Lausanne).

[ref-20] Chou Jonathan, Lin Jeffrey H., Brenot Audrey, Kim Jung-whan, Provot Sylvain, Werb Zena (2013). GATA3 suppresses metastasis and modulates the tumour microenvironment by regulating microRNA-29b expression. Nat Cell Biol.

[ref-21] Bonaventura Paola, Shekarian Tala, Alcazer Vincent, Valladeau-Guilemond Jenny, Valsesia-Wittmann Sandrine, Amigorena Sebastian, Caux Christophe, Depil Stéphane (2019). Cold Tumors: A Therapeutic Challenge for Immunotherapy. Front Immunol.

[ref-22] Ferronika Paranita, Dhiyani Safira Alya, Budiarti Tri, Widodo Irianiwati, Rinonce Hanggoro Tri, Anwar Sumadi Lukman (2022). Regulatory T Cells but Not Tumour-Infiltrating Lymphocytes Correlate with Tumour Invasion Depth in Basal Cell Carcinoma. Diagnostics (Basel).

[ref-23] Chiang Elizabeth, Stafford Haleigh, Buell Jane, Ramesh Uma, Amit Moran, Nagarajan Priyadharsini, Migden Michael, Yaniv Dan (2023). Review of the Tumor Microenvironment in Basal and Squamous Cell Carcinoma. Cancers (Basel).

[ref-24] Ho I.-Cheng, Tai Tzong-Shyuan, Pai Sung-Yun (2009). GATA3 and the T-cell lineage: essential functions before and after T-helper-2-cell differentiation. Nat Rev Immunol.

[ref-25] Zhang Qixin, Qi Tiezheng, Long Yu, Li Xiaowen, Yao Yiyan, Wu Qi, Zou Anrong, Qthmane Belaydi, Liu Peihua (2022). GATA3 Predicts the Tumor Microenvironment Phenotypes and Molecular Subtypes for Bladder Carcinoma. Front Surg.

[ref-26] Omland Silje H., Nielsen Patricia S., Gjerdrum Lise M. R., Gniadecki Robert (2016). Immunosuppressive Environment in Basal Cell Carcinoma: The Role of Regulatory T Cells. Acta Derm Venereol.

[ref-27] Fujimura Taku, Kakizaki Aya, Kambayashi Yumi, Aiba Setsuya (2012). Basal cell carcinoma with spontaneous regression: a case report and immunohistochemical study. Case Rep Dermatol.

[ref-28] Wong D. A., Bishop G. A., Lowes M. A., Cooke B., Barnetson R. S., Halliday G. M. (2000). Cytokine profiles in spontaneously regressing basal cell carcinomas. Br J Dermatol.

[ref-29] Cohen Helit, Ben-Hamo Rotem, Gidoni Moriah, Yitzhaki Ilana, Kozol Renana, Zilberberg Alona, Efroni Sol (2014). Shift in GATA3 functions, and GATA3 mutations, control progression and clinical presentation in breast cancer. Breast Cancer Res.

